# Otalgia and eschar in the external auditory canal in scrub typhus complicated by acute respiratory distress syndrome and multiple organ failure

**DOI:** 10.1186/1471-2334-11-79

**Published:** 2011-03-30

**Authors:** Bor-Jen Lee, Chia-Yi Chen, Sung-Yuan Hu, Yu-Tse Tsan, Tzu-Chieh Lin, Lee-Min Wang

**Affiliations:** 1 Department of Emergency Medicine, Taichung Veterans General Hospital, Taichung City, 00407, Taiwan; 2 Intensive Care Unit, Department of Internal Medicine, Taichung Veterans General Hospital, Taichung City, 00407, Taiwan; 3 Department of Emergency Medicine, Chung Shan Medical University Hospital, Taichung City, 40201, Taiwan; 4 School of Medicine, Chung Shan Medical University, Taichung City, 40201, Taiwan; 5 School of Nutrition and Institute of Nutritional Science, Chung Shan Medical University, Taichung City, 40201, Taiwan; 6 National Taichung Nursing College, Section 1, San Min Road, Taichung City, Taiwan; 7 National Taiwan University, Taipei, 10617, Taiwan, China

## Abstract

**Background:**

Scrub typhus, a mite-transmitted zoonosis caused by *Orientia tsutsugamushi*, is an endemic disease in Taiwan and may be potentially fatal if diagnosis is delayed.

**Case presentations:**

We encountered a 23-year-old previously healthy Taiwanese male soldier presenting with the right ear pain after training in the jungle and an eleven-day history of intermittent high fever up to 39°C. Amoxicillin/clavulanate was prescribed for otitis media at a local clinic. Skin rash over whole body and abdominal cramping pain with watery diarrhea appeared on the sixth day of fever. He was referred due to progressive dyspnea and cough for 4 days prior to admission in our institution. On physical examination, there were cardiopulmonary distress, icteric sclera, an eschar in the right external auditory canal and bilateral basal rales. Laboratory evaluation revealed thrombocytopenia, elevation of liver function and acute renal failure. Chest x-ray revealed bilateral diffuse infiltration. Doxycycline was prescribed for scrub typhus with acute respiratory distress syndrome and multiple organ failure. Fever subsided dramatically the next day and he was discharged on day 7 with oral tetracycline for 7 days.

**Conclusion:**

Scrub typhus should be considered in acutely febrile patients with multiple organ involvement, particularly if there is an eschar or a history of environmental exposure in endemic areas. Rapid and accurate diagnosis, timely administration of antibiotics and intensive supportive care are necessary to decrease mortality of serious complications of scrub typhus.

## Background

Scrub typhus, a mite-transmitted zoonosis caused by *Orientia tsutsugamushi*, is an endemic disease in Taiwan and may be potentially fatal if diagnosis is delayed. It is an acute febrile illness characterized by a typical necrotic primary lesion (eschar), generalized lymphadenopathy, maculopapular skin rash, and nonspecific symptoms and signs. The incidence of eschar reported in patients with scrub typhus is low, < 25%, and only 5% are found over head, face and neck. An eschar in the external auditory canal has not been reported previously, so the diagnosis of scrub typhus was delayed with serious complications, including acute respiratory distress syndrome (ARDS) and multiple organ failure (MOF). In patients with scrub typhus, the incidence and mortality rate of ARDS are 11.1%-15.2% and 20%-25%, respectively. Reported here is a case of an eschar in the external auditory canal in scrub typhus complicated by ARDS and MOF in a 23-year-old previously healthy Taiwanese male soldier.

## Case Presentation

**Figure 1:**
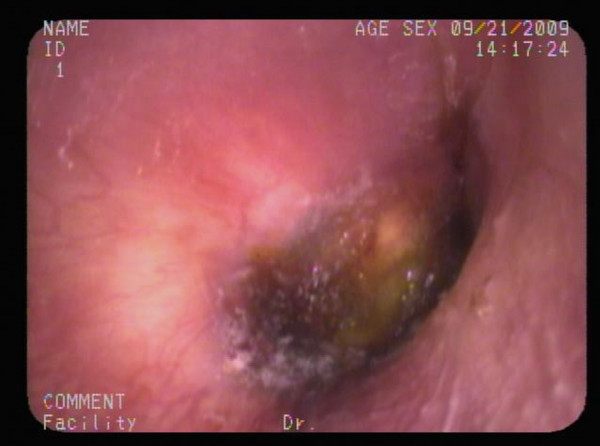
**Eschar, a slightly raised erythema surrounding a black necrotic center, in right external auditory canal, centered at 6 o'clock**.

**Figure 2:**
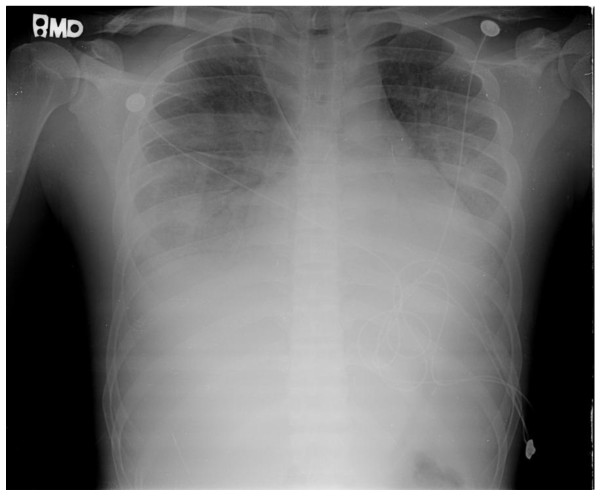
**Chest x-ray revealed bilateral diffuse infiltration disclosing a presentation of acute respiratory distress syndrome**.

A 23-year-old previously healthy Taiwanese male soldier presented with right ear pain and an eleven-day history of intermittent high fever, up to 39°C, after training in the jungle. He was admitted to a local clinic and amoxicillin/clavulanate 1 g was prescribed every 6 hours for otitis media, but the fever persisted. Furthermore, he developed a maculopapular skin rash over whole body and cramping abdominal pain with watery diarrhea appeared on the sixth day of fever. He was referred to our institution as his clinical condition has deteriorating with progressive dysponea and cough in the 4 preceding days. Physical examination showed an appearance of cardiopulmonary distress, icteric sclera, an eschar (Figure 1) in the right external auditory canal and bilateral basal rales. Laboratory evaluation revealed a white blood cell count of 9100/mm^3 ^(reference range [RR]: 4000-11000/mm^3^) with 87.9% segmented neutrophils, hemoglobin 14.6 g/dL (RR: 14-16 g/dL), platelet counts 24 × 10^3^/mm^3 ^(RR: 140-400 × 10^3^/mm^3^), blood urea nitrogen 25 mg/dL, creatinine 1.5 mg/dl (RR: 0.7-1.4 mg/dL), sodium 140 mEq/L, potassium 3.9 mEq/L, chloride 108 mEq/L, calcium 8.2 mg/dL, total protein 5.8 g/dL (RR: 6.0-8.0 g/dL), albumin 3 g/dL (RR: 3.5-5.0 g/dL), total bilirubin 4.8 mg/dL (RR: 0.1-1.2 mg/dL), direct bilirubin 2.7 mg/dL (RR: 0.0-0.2 mg/dL), C-reactive protein 16.23 mg/dL (RR <0.3 mg/dL), aspartate aminotransferase (AST) 368 IU/L (RR: 8-38 IU/L), alanine aminotransferase (ALT) 271 IU/L (RR: 4-44 IU/L), alkaline phosphatase (ALK) 324 IU/L (RR: 50-190 IU/L), lactate dehydrogenase 783 IU/L (RR: 120-240 IU/L), glucose 94 mg/dL, creatine phosphokinase 229 IU/L (RR: 10-160 IU/L), and a positive for Weil-Felix reaction with a *Proteus OX-K *titer of 1:1280 on day 11 of fever. Arterial blood gas analysis was pH 7.501, PaCO_2 _38.1 mmHg, PaO_2 _76 mmHg, HCO_3_^- ^30.1 mmol/l, and BEB 7.2 with a FiO_2 _of 60%. A central venous line was setup for monitoring his central venous pressure and fluid replacement because of hypotension. Chest x-ray (Figure 2) revealed bilateral diffuse infiltration. An endotracheal intubation was performed due to progressive dyspnea and desaturation, and then he was admitted to the intensive care unit. Doxycycline of 100 mg was prescribed every 6 hours for a clinical therapeutic trial of scrub typhus with ARDS and MOF. Fever subsided dramatically the next day and the endotracheal tube was removed on day 3. Chest x-ray abnormalities resolved gradually and full blood count and biochemistry results returned to normal ranges without sequelae. He was discharged on day 7 with tetracycline 500 mg every 6 hours for 7 days.

## Discussion

Scrub typhus, a mite-transmitted zoonosis caused by the intracellular Gram-negative bacteria, *Orientia tsutsugamushi *(previously known as *Rickettsia tsutsugamushi*), is a disease distributed throughout the Asia Pacific rim and endemic in South Korea, China, Japan, Taiwan, Pakistan, India, Thailand, Malaysia and northern Australia. It is an acute febrile illness characterized by a typical necrotic primary lesion (eschar), generalized lymphadenopathy, maculopapular skin rash, and nonspecific symptoms and signs such as fever, chills, headache, alerted sensorium, seizure, sorethroat, otalgia, cough, dysponea, vomiting, abdominal pain, jaundice, diarrhea, hepatosplenomegaly, abnormal bleeding, arthralgia and myalgia[1-12]. Unfortunately the typical eschar and skin rash are found on examination in 46%~92% of cases in Korea and less than 10% of cases in Thailand[4,12]. Serious complications, including encephalitis, meningitis, pericarditis, myocarditis, cardiac arrhythmia, interstitial pneumonia, ARDS, acute renal failure, acute hepatic failure, acute hearing loss and septic shock, generally occur in the second week of the course and may be potentially fatal if there is a delay in diagnosis and treatment[1-12]. Most cases of scrub typhus have a delayed (or no) diagnosis because the history of travel or exposure history is omitted and the pathognomonic eschar of scrub typhus is not found or not adequately searched for in patients with fever of unknown origin[1-12]. The incidence of eschar, a slightly raised erythema surrounding a black necrotic center, over head, face and neck is 5%[4]. In our present case, he was a solider and was on training in the jungle, so his ear was often close to the ground providing the mites with an opportunity to enter the ear canal and attach causing the resultant eschar. Although the eschar may be painless and otalgia is an unusual manifestation, there was an incidence of 19% in scrub typhus with otalgia and one case of acute sensorineural hearing loss has been reported[7]. Initially, he was treated as otitis externa at a local clinic because of fever, otalgia, and an unusual and undetectable eschar in the external auditory canal, resulting in delayed diagnosis and treatment of scrub typhus complicated with ARDS and MOF with respiratory, renal, hepatic and hematologic involvement in our case. He was referred to our institution and scrub typhus was strongly suspected based on clinical exposure history, eschar in the external canal on physical examination and multiple organ involvement, so clinical therapeutic doxycycline was prescribed and the fever subsided dramatically. The pulmonary manifestations of scrub typhus include varying grades of bronchitis and interstitial pneumonitis progressing to ARDS. ARDS is a rarely reported but is a serious complication of scrub typhus with an incidence of 11.1%-15.2%. Only two short communication reports of scrub typhus complicated by ARDS have been published[2,3]. Acute renal failure is due to acute tubular necrosis caused by direct invasion of *Orientia tsutsugamushi *[6,12]. Abnormal laboratory evaluations include leukopenia (19%) or leukocytosis (6%-34%), thrombocytopenia (44%-100%), elevation of AST (81%-100%), ALT (75%-100%), ALP (84.2%-100%) and total bilirubin (38.5%-100%), hypoalbuminemia (83.3%), and increased creatinine (9%)[1-3,5,6,8-10,12]. Weil-Felix test has low sensitivity and specificity to the diagnosis of scrub typhus, but it can be a useful tool when used and interpreted in the correct clinical context. The criterion for a positive result is either one determination of a titer of 1:320 or greater or a four-fold rise in titer starting from 1:50[10-12]. Weil-Felix test with *Proteus OX-K *titer of 1:1280 was obtained on day 11 after exposure in our case. While all current scrub typhus tests have limitations and the Weil-Felix and immunofluorescent antibody (IFA) tests have low sensitivity and specificity[11]. Weil Felix was the only test available at our institution. Dyspnea, cough, white blood cell counts, hematocrit, platelet, total bilirubin, older age, chronic obstructive pulmonary disease, hypoalbuminemia, and prolonged prothrombin time, presence of early pneumonitis and delayed used of appropriate antibiotics have been reported as significant predictors of ARDS[2,3,12]. The mortality rate was 6.1%-30% in previous literature and the major cause of mortality was delay in diagnosis and treatment[1-6,12]. Scrub typhus was diagnosed based on the clinical picture and the rapid response to doxycycline in our case. In ICU, doxycycline for scrub typhus and ventilator support for ARDS were prescribed, so the clinical condition of this patient with multiorgan involvement, including respiratory, liver, renal and hematological involvement, improved gradually with a full course of doxycycline in this patient.

## Conclusion

We recommend that scrub typhus should be taken into consideration in acutely febrile patients with varying degree of respiratory distress, impaired liver function, acute renal failure and hematologic involvement, particularly if there is an eschar or a history of environmental exposure in an endemic area[1-9,11,12]. Rapid and accurate diagnosis, timely administration of appropriate antimicrobial therapy and intensive supportive care are crucial for the recovery of ARDS and other serious complications of scrub typhus[1-3,5-9,12].

## Competing interests

The authors declare that they have no competing interests.

## Authors' contributions

BJL took care of this patient in the intensive care units. CYC drew up the first draft of the report. SYH made a substantial contribution to draft the manuscript and revised the draft all over the course of submission. All authors read and approved the final manuscript.

## Consent

Written informed consent was obtained from the patient for publication of this study. A copy of the written consent is available for review by the Editor-in-Chief of this journal.

## Pre-publication history

The pre-publication history for this paper can be accessed here:

http://www.biomedcentral.com/1471-2334/11/79/prepub
